# Is Obstructive Sleep Apnea-Associated Adenoid Hypertrophy Linked to Scoliotic Attitudes in Children?

**DOI:** 10.7759/cureus.47307

**Published:** 2023-10-19

**Authors:** Fatih Ugur, Kubra Topal, Mehmet Albayrak, Murat Topal

**Affiliations:** 1 Orthopedics and Traumatology, Faculty of Medicine, Kastamonu University, Kastamonu, TUR; 2 Otolaryngology - Head and Neck Surgery, Private Practice, Kastamonu, TUR; 3 Orthopedics and Traumatology, Ozel Tekirdag Yasam Hospital, Tekirdag, TUR

**Keywords:** scoliotic attitude, prevalence, obstructive sleep apnea, early onset scoliosis, adenoidectomy

## Abstract

Introduction

Scoliosis, a multifaceted spinal deformity commonly affecting pediatric and adolescent populations, has spurred extensive scientific inquiry to understand its origins and impacts. Early-onset scoliosis (EOS), characterized by spinal curvature exceeding 10° before the age of 10, presents a unique challenge necessitating a comprehensive understanding of its etiological factors. Within this context, the potential role of hypoxia-induced by adenoid hypertrophy in contributing to the pathogenesis of EOS has emerged as an intriguing avenue of investigation.

Materials and methods

This retrospective study was conducted focusing on radiological and clinical data pertaining to children below 10 years of age who underwent isolated adenoidectomy for adenoid hypertrophy. Preoperative posteroanterior standing chest radiographs were utilized for scoliosis assessment, with Cobb angles serving as the primary measurement metric. To ensure accuracy and reliability, Cobb angle measurements were independently performed by two experienced observers. Statistical analyses encompassed the Mann-Whitney U test, Spearman correlation analysis, and intraclass correlation coefficient calculations to evaluate interobserver agreement.

Results

Among the cohort of 218 pediatric adenoidectomy patients, 177 individuals had radiographs suitable for EOS evaluation. The mean age of the participants was 5.72±2 years, with a nearly equal distribution of 52.5% male and 47.5% female patients. Strikingly, the study identified a 10.2% prevalence of coronal plane curvatures exceeding the critical threshold of 10°, indicative of EOS. The robust interobserver reliability was demonstrated by a commendable mean interclass correlation coefficient (ICC) value of 0.926, affirming consistent and accurate Cobb angle measurements between the observers.

Conclusion

In light of the heightened prevalence of EOS observed in children undergoing adenoidectomy, this study provides a compelling impetus for exploring the potential interrelationship between adenoid hypertrophy, hypoxia, and the emergence of early-onset scoliosis. The study underscores the importance of prospective research to elucidate the complex mechanisms connecting these factors, offering insights into potential risk factors and underlying pathogenic pathways associated with the development of early-onset scoliosis.

## Introduction

Scoliosis, a notable spinal deformity prominently observed in pediatric and adolescent populations, represents a complex three-dimensional distortion of the spinal axis and trunk. The classification of scoliosis is based on parameters such as age of onset, etiology, severity, and curvature configuration [1]. However, existing classification systems tailored for adolescents and adults exhibit limitations in comprehensively addressing the intricate pathophysiology characteristic of early-onset scoliosis (EOS) [2]. EOS was used to confirm a worse prognosis in scoliosis beginning before the age of 10 in comparison with scoliosis developing from age 10 years and beyond, known as late-onset scoliosis (LOS) [3]. Although the term originated in the 20th century, there were disagreements on how to best define this condition until a consensus statement was released by the Scoliosis Research Society's Growing Spine Committee, alongside the development of the classification of EOS [3,4]. Collectively, leading experts in EOS concur that the management principles are uniform for patients under the age of 10 years, while these principles diverge for LOS [4]. EOS is characterized by any spinal attitude exceeding 10° in children under the age of 10 years with five primary etiological categories mentioned as follows: congenital, neuromuscular, syndromic, thoracogenic, and idiopathic [2-4]. In spite of recent progress, there have been difficulties in ascertaining the occurrence and frequency of EOS [4]. In the context of epidemiological assessment, significant challenges arose due to the considerable variability in defining age groups and the disparities in determining suitable causative classifications. These challenges hindered the attainment of a cohesive and standardized epidemiological evaluation [4,5].

The significance of scoliotic attitude in public health and healthcare infrastructure is paramount, prompting extensive assessments of prevalence and early detection of influencing factors to mitigate its profound consequences [5]. Although EOS is associated with high rates of pulmonary insufficiency, which is believed to contribute significantly to mortality and morbidity in patients [3], it is essential not to overlook the potential for non-progression and therefore resolution, particularly in the predominantly idiopathic type [4].

In several research investigations, the role of hypoxia is postulated as a potential contributing factor to the development of scoliosis. A significant portion of patients, particularly those with idiopathic, neurological, and congenital scoliosis, exhibited deviations in arterial oxygen saturation and carbon dioxide levels [6].

Additionally, in certain animal studies, teratogenic effects associated with congenital scoliosis resulting from prenatal exposure to hypoxia have been observed. This exposure has been shown to lead to vertebral anomalies, ultimately culminating in spinal deformities [7].

Recently, there has been increasing recognition of the pivotal role played by hypoxia-inducible factor (HIF) as a crucial molecular component responsible for cellular oxygen sensing and adaptive responses. Furthermore, there have been studies elucidating the production of HIF and structural alterations within the nucleus pulposus when exposed to hypoxic conditions [8].

In the context of identifying causative factors to mitigate the progression of curvature in idiopathic cases of scoliosis, the impact of HIF dysregulation during hypoxic stress in adolescents with idiopathic scoliosis is considered of significant importance. It is also emphasized that this dysregulation can have effects on muscle tonicity in the paraspinal muscles located at the apex of the curvature [9].

Given the implications of these findings, the involvement of hypoxia in spinal dynamics, particularly in the context of EOS, warrants comprehensive investigation. Traditionally, obstructive sleep apnea (OSA) in children, with a reported prevalence ranging from 1% to 5% and peaking between ages two and eight years, was treated with adenotonsillectomy, but recent findings suggest that adenoidectomy alone may suffice, as untreated OSA can lead to intermittent hypoxia, exacerbating its impact on EOS [10-13].

In this study, we hypothesized that children undergoing isolated adenoidectomy alone could experience intermittent hypoxia until the time of the surgery, which is potentially linked to the induction of an inflammatory response and an increased incidence of spinal curvature. Consequently, this investigation aims to elucidate the prevalence of EOS in children undergoing adenoidectomy.

## Materials and methods

This retrospective analysis was granted ethical approval by the institutional review board under reference number 2023-KAEK-4 and was exclusively centered on the Kastamonu Training and Research Hospital. The study involved the examination of radiological and clinical data related to children under the age of 10 years who had undergone adenoidectomy as a standalone procedure. The assessment encompassed patients who had undergone adenoidectomy alone between January 2017 and December 2022. Comprehensive data, including gender, age, and Cobb angles, were meticulously collected.

The preoperative assessment for adenoidectomy involved the acquisition of standing posteroanterior (PA) chest radiographs, similar to those employed in spinal curvature assessments, to evaluate the preoperative conditions. In this context, scoliosis evaluation data were derived from radiographs taken during pediatric lung imaging, which included wide-angle images that deviated from the established protocol, and in this manner, the radiographs that inadvertently transformed into all vertebral column images instead of chest radiographs were assessed. Only patients with radiographs covering both thoracic and lumbar vertebral regions, appropriately aligned shoulders, a natural head position, and suitability for scoliotic attitude assessment were included. Patients with radiographs that had the potential to yield incorrect assessments due to factors such as incomplete visualization of the entire vertebral column or the presence of pseudoscoliosis-like patterns caused by pelvic obliquity were excluded from the study.

To assess vertebral curvatures in pediatric adenoidectomy patients and determine the incidence of EOS, Cobb angle measurements were conducted using a digital angle measurement tool. Prior to measurements, all observers received comprehensive training in utilizing the picture archiving and communication system (PACS) database, performing digital Cobb angle measurements, and documenting their findings accurately.

Cobb angles, which quantify coronal plane vertebral deformities on PA radiographs by measuring the angle between the superior endplate of a superior-tilted vertebra and the inferior endplate of an inferior-tilted vertebra, were assessed for each patient. To enhance measurement accuracy and reliability, two orthopedic surgeons independently conducted measurements at separate times, and the lowest Cobb angle value was considered [2,4].

To ensure the validity and consistency of Cobb angle measurements across different observers, an evaluation of interobserver reliability was conducted by calculating intraclass correlation coefficients. The primary objective of this thorough preoperative assessment was to ascertain the prevalence of EOS within the study population.

Statistical analysis

Data distribution was examined using the Shapiro-Wilk test, which assesses whether the data follows a normal pattern. For comparing two independent groups with non-normal data, the Mann-Whitney U test was employed, a method that does not rely on normal distribution assumptions. Spearman correlation analysis was used to assess the relationship between numerical variables, a technique known for measuring associations without assuming linear relationships. To evaluate the agreement among observers, interclass correlation coefficient (ICC) was calculated - an established metric for understanding the consistency of observers' assessments. Numerical data were described using medians, while categorical data were presented as percentages to offer a comprehensive view. All these statistical analyses were performed using IBM SPSS Statistics version 26.0 software (Armonk, NY: IBM Corp.), with the significance level set at α=0.05, indicating noteworthy findings at a 95% confidence level.

## Results

**Figure 1:**
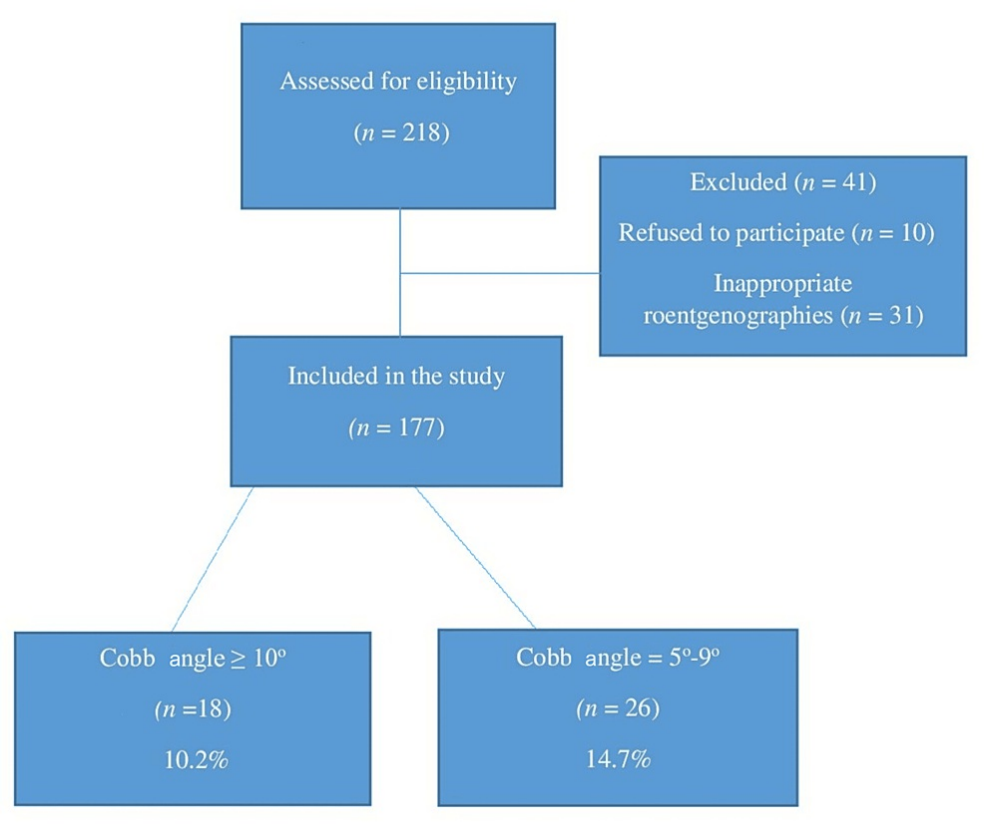
Flowchart depicting the distribution based on the percentage of patient participation and Cobb angle values.

This study enrolled 218 pediatric adenoidectomy patients. Ten of the patients refused to participate in the study. Thirty-one patients were excluded from the study due to inappropriate roentgenographies, either because of pelvic obliquity or the vertebral column not being fully visible. A total of 177 patients’ chest radiographs were suitable for the evaluation of scoliotic curvature. The mean age of the children was 5.72±2 years, with an age range spanning from two to nine years. Among the participants, 93 (52.5%) were male, and 84 (47.5%) were female. The flowchart of the study is shown in Figure 1.

In a sample of 177 patients, 18 individuals (10.2%) were classified as having EOS based on a Cobb angle measurement of 10° or higher, as determined through an analysis of patient records. However, there was no available information regarding the underlying cause of EOS in these 18 patients. Among these 18 patients, two (11%) were male while the remaining 16 (89%) were female. Furthermore, it was observed that patients in the age range of three to eight years, with an average age of 5.7 years, exhibited Cobb angle values ranging from 10° to 17°, with an average Cobb angle of 12.8°. A total of 26 patients (14.7%) had Cobb angles in the range of 5° to 9° (average 6.4°). Among the 26 patients evaluated, who were between three and nine years old (average 5.3), there were 13 girls and 13 boys.

In the children included in the study, a statistically significant relationship between age and the accepted Cobb angle could not be found (p=0.450). When evaluated according to gender, the Cobb angle has a statistically significant difference (p=0.007), and the Cobb angle is found to be significantly higher in girls compared to boys. Additionally, 133 patients demonstrated normal X-rays without significant curvatures.

**Figure 2:**
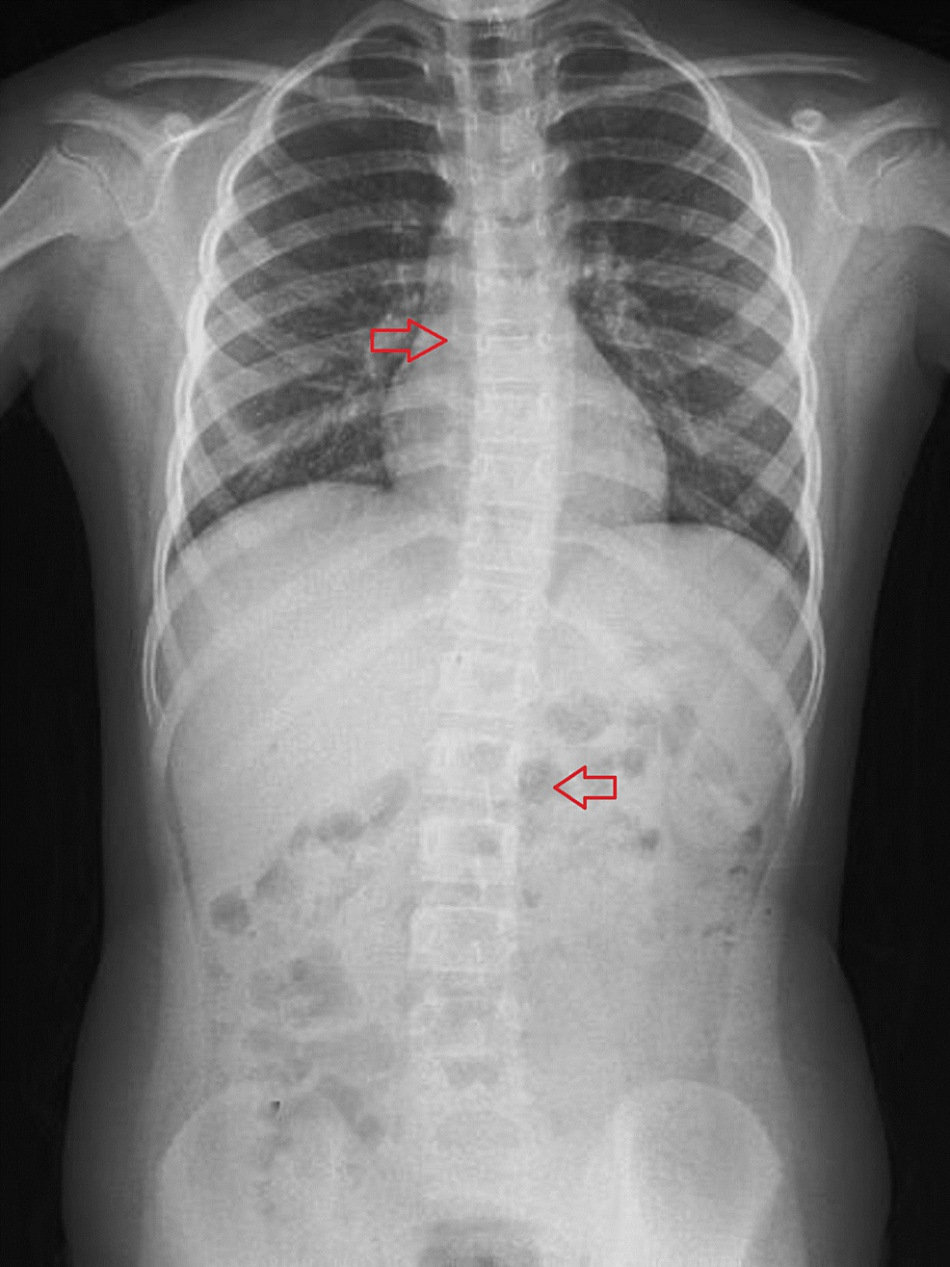
Roentgenography of an early-onset scoliosis (EOS) patient. Left and right arrows indicate the scoliotic vertebrae.

**Figure 3:**
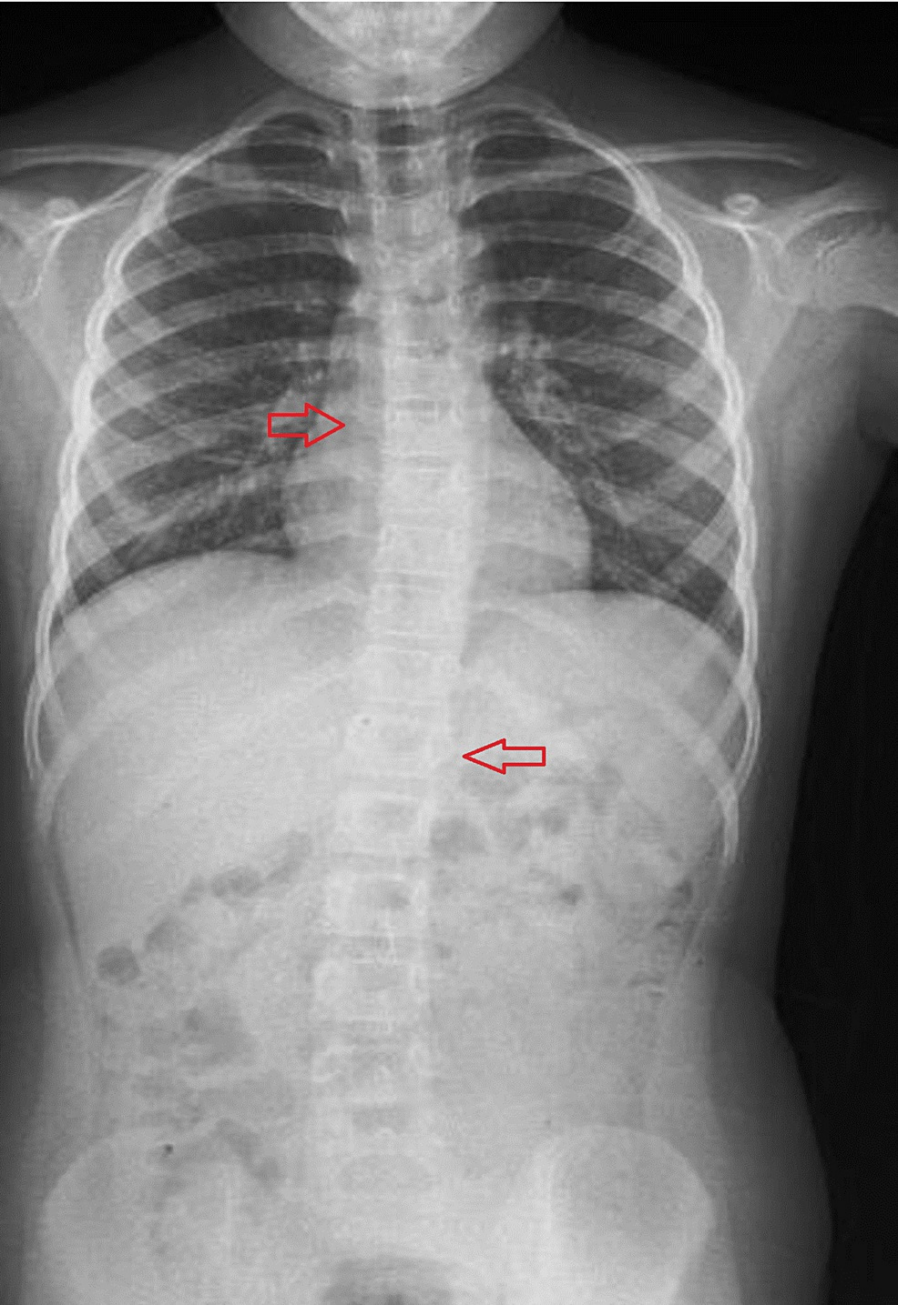
Roentgenography of another patient with early-onset scoliosis (EOS). Left and right arrows indicate the most tilted vertebrae.

The subsequent analysis of interobserver reliability unveiled a notable average ICC of 0.926, serving as a noteworthy demonstration of the coherent agreement between the observers regarding their interpretation of the Cobb angles. Roentgenographies of two patients who underwent preoperative chest radiographies for adenoidectomy surgery are shown in Figures 2, 3. EOS is observed in the radiographies.

## Discussion

In cases where adenoid hypertrophy leads to upper airway obstruction severe enough to require surgery, including children under the age of 10 years, a significantly higher prevalence of 10.2% was observed in the assessment of scoliotic attitudes [2-4]. This prevalence varies considerably across different studies, with recent research reporting a rate as low as 0.077%. Based on findings from different studies, idiopathic EOS constitutes a relatively low percentage of all cases of idiopathic scoliosis, with prevalence rates ranging from less than 1% to approximately 0.13% [4]. It is worth noting that these studies specifically focused on the idiopathic category. When considering the reported prevalence studies in this context, it is observed that scoliotic postures are significantly prevalent in patients undergoing adenoidectomy alone due to adenoid hypertrophy. This notable difference in prevalence raises questions about whether EOS has an etiological factor, particularly in cases of intermittent hypoxia resulting from OSA, a common indication for adenoidectomy [10-13].

In the noteworthy study conducted by MacKintosh et al. on scoliosis and sleep abnormalities, they identified a significant association between sleep-disordered breathing and hypoxia in children with EOS. Their research revealed a remarkably high prevalence of sleep-disordered breathing, affecting 40.9% of the participants. Interestingly, within this group of patients, 8.1% underwent adenotonsillectomy operations, further emphasizing the remarkable findings of their study [14].

Adenoid hypertrophy, a significant risk factor for OSA requiring adenoidectomy as primary treatment, should be considered alongside other factors like obesity, anatomical issues, ethnicity, and upper airway collapsibility, as these factors can contribute to residual OSA following unsuccessful surgical treatment [10-13]. Epidemiological studies have shown that the prevalence of adenoid hypertrophy varies between 19% and 38% among children aged three to nine years [15]. To address the high likelihood of adenoid hypertrophy, adenoidectomy has been reported to have the highest success rate at 1.2% [13]. Similarly, OSA is reported to have a prevalence rate of 1-5%, which is comparable to the prevalence of idiopathic scoliosis, also around 5% [1,10]. These findings suggest that these conditions are relatively common in the population, and further research should be conducted to better understand their prevalence and underlying causes.

It is noteworthy that these conditions can affect individuals under the age of 10 years, even though they are more commonly associated with individuals over 10 years of age. This implies that the onset of these conditions may occur before the age of 10 years, particularly in the case of idiopathic scoliosis. Therefore, it is important to pay attention to the possibility of these conditions developing in younger age groups.

OSA, regardless of its cause, involves recurring episodes of apnea and hypopnea, leading to intermittent hypoxemia (IH), changes in intrathoracic pressure, and disrupted sleep patterns. These factors contribute to the mortality and morbidity associated with OSA [10]. Both animal and human studies provide strong evidence linking IH to harmful effects at the tissue level. IH induces oxidative stress by increasing the production of reactive oxygen species, stimulates angiogenesis, and leads to heightened sympathetic activity, resulting in elevated blood pressure and systemic inflammation. These factors collectively contribute to various chronic health problems and an increased risk of mortality, including cardiovascular disease, metabolic dysfunction, and cognitive decline [10,16]. It is not yet clear whether OSA has an impact on vertebral development in growing children, despite its significant implications for pediatric health.

Hypoxia effects are observed not only in OSA but also in adenoid hypertrophy [16]. When comparing preoperative and postoperative states in adenoidectomy, a significant and statistically meaningful decrease in postoperative serum total oxidant level values has been observed, indicating a reduction in the effects of hypoxia [17]. Nanduri et al. emphasized the importance of the duration of exposure to hypoxia, stating that short-term intermittent hypoxia exposure results in complete correction shortly after the exposure is removed, in contrast, long-term exposure leads to persistent abnormalities even after the cause has been eliminated [18]. This study, particularly the relationship between adenoid hypertrophy and OSA, highlights the need to minimize the effects of intermittent hypoxia and even consider early intervention in addressing scoliotic attitudes.

It is observed that a hypoxic environment leads to complex structural and cellular changes. In a hypoxic environment, homeostasis is orchestrated by the modulating influences of HIFs, primarily HIF-1α and HIF-2α [9,19]. As articular cartilage remains chronically hypoxic, the expression of HIF proteins is consistently high within chondrocytes, impacting their transcriptional response to other stimuli. In hypoxic conditions, changes occur in the cartilage. HIF-1α promotes the deposition of matrix components such as type II collagen. HIF-2α triggers a chondroanabolic stimulus, enhancing the matrix deposition of dedifferentiated chondrocytes [19]. Notably, Tam et al. elaborated on the maladaptive expression and regulatory dysregulation of HIF-2α as a potential pathogenic factor in the development of idiopathic scoliosis [9].

Ongoing efforts connect idiopathic scoliosis emergence with hypoxia, notably reduced oxygen availability [9]. Hypoxia's impact extends to the annulus fibrosus and nucleus pulposus, with HIF-1α and HIF-2α regulation [19]. Liu et al. demonstrated that chronic intermittent hypobaric hypoxia in rats led to increased expression of bFGF, TGFβ1, and HIF-1α. Collagen I and II levels increased, and the nucleus pulposus expanded, constituting over half the disk volume [8]. Jiang et al. reported significant muscle volume and fatty infiltration disparities in adolescent idiopathic scoliosis (AIS) patients, indicating hypoxia's impact on adipose tissue dynamics [20].

It becomes apparent that hypoxia's impact extends beyond the confines of congenital scoliosis, also permeating the realm of idiopathic scoliotic attitude [9]. Therefore, we propose that frequent early childhood pathology, adenoid hypertrophy, causing OSA and intermittent hypoxia, could correlate with EOS. In this study, 10.2% of patients were diagnosed with EOS, and 14.7% had coronal plane deformities of less than 10°. Comparing our findings with previous prevalence studies, the incidence rate in children undergoing adenoidectomy appears notably elevated [4]. This outcome emphasizes the need to thoroughly explore the potential involvement of adenoid hypertrophy-induced hypoxia in EOS etiology.

While EOS patients encompass a spectrum of etiological factors, leading to severe progression and high mortality and morbidity rates [3], with males showing notably higher comorbidity rates than females [21], AlNouri et al. revealed that not all EOS cases exhibit progression [4]. The rate of non-progression among all EOS cases was found to be 44.3%. Notably, in idiopathic cases, scoliosis resolution was more common than progression [4].

Various studies have reported different male-to-female ratios. In Karpe et al.'s study focusing on LOS, they found that curves exceeding 10° affect nearly equal numbers of males and females. However, when the curves exceed 30°, the ratio of affected females to males increases to 10 to 1 [5]. Consistent with AlNouri et al.'s findings, in our study it is also observed a higher proportion of females in cases of spinal curvature measuring 10° or more [4].

Scoliosis diagnosis relies on a comprehensive understanding of clinical and radiological parameters, with the Cobb angle serving as a fundamental metric [22]. Notably, for curves exceeding 20°, clinical assessment demonstrates exceptional sensitivity and specificity, reaching 90%. However, its accuracy tends to diminish for curves measuring below 20° [23]. Our study highlights a robust correlation between clinical and radiological data in assessing scoliotic deformities. Our study also indicates that data with Cobb angles below 20° are unlikely to provide sufficient clinical evaluation information. In our study, it was evident that having sufficient data for clinical assessment, especially from an etiological perspective, was crucial. Unfortunately, we were unable to obtain data related to the etiological causes in our study.

To enhance diagnostic precision, we explored the utility of a scoliometer with a 5° referral threshold, demonstrating promise in detecting approximately 87% of cases within the 10-20° Cobb angle range, rising to 100% for angles exceeding 20° [24]. These findings suggest the valuable role of clinical assessment alongside radiological evaluation.

It is vital to acknowledge potential limitations associated with Adam's forward-bending test as an early diagnostic criterion for detecting scoliotic attitudes [25]. The existence of Cobb angles ranging from 10 to 17° within our dataset underscores that clinical data alone may not be fully supported, particularly in this mild range of deformities, which may not significantly impact patient diagnosis.

Debates persist concerning the use of chest radiographs in evaluating scoliotic attitudes [26]. Pan et al. reported a 14.9% prevalence of scoliotic curves in patients aged 4-18 years who underwent strabismus surgery, relying solely on chest radiographs without lumbar imaging [27]. In the study conducted by Pan et al., the high proportion of cases with Cobb angles of 20° or less in the prevalence of scoliosis suggests support for an increased rate of thoracic vertebral scoliosis in scoliosis cases evaluated through chest radiographs. While these results highlight the potential of chest radiographs in diagnosing scoliosis, the suitability of this approach for precise scoliotic attitude assessment remains a subject of ongoing discussion [26].

Our study exclusively incorporated preoperative standing radiographs, meticulously selected to ensure their suitability for assessing scoliotic attitudes. Pediatric chest radiography often faces notable discrepancies between recommended and actual imaging fields, with the current field area being up to 2.8 times larger than the ideal field. Factors contributing to these disparities encompass exposure techniques, patient positioning, the patient's ability to maintain the position, and the accuracy of field collimation [28].

While OSA, which is frequently observed in up to 5% of individuals under the age of 10 years, is known to have effects on lung and heart disease due to intermittent hypoxia, its impact on idiopathic scoliosis, which is observed at a similar rate, is not known. Considering the public health implications, both of these conditions should be examined closely. OSA, in particular, is associated with intermittent hypoxia, which can lead to heart and lung diseases [22]. Importantly, our study unveils how adenoid hypertrophy, a common cause often treated with adenoidectomy for OSA, can induce vertebral column issues in pediatric patients due to intermittent hypoxia.

The relationship between hypoxia and scoliosis has been the subject of extensive research, starting with Shaw and Read and continuing with investigations into the role of HIFs [6]. This study uniquely demonstrates the co-occurrence of scoliosis in OSA cases arising from adenoid hypertrophy, highlighting the potential interplay between intermittent hypoxia and scoliosis pathogenesis. Our findings emphasize the importance of early diagnosis and intervention, particularly in cases where adenoid hypertrophy is severe enough to warrant surgery. Early recognition is paramount, given the documented influence of conditions such as axial loading on scoliosis progression, as indicated in the progression models discussed by Hawes et al. [29]. Although the multifactorial etiological causes of this widespread disease, which can still not be fully understood, suggest that progression can be controlled through preventive measures, screening programs for this disease are recommended to be carried out after the age of 10 years worldwide. However, our study has shown that scoliosis screening programs can also be important for individuals under the age of 10 years [30]. These screening programs should not only assess scoliosis but also consider the evaluation of OSA [10]. Furthermore, it is essential that these individuals are subsequently evaluated by orthopedic specialists as well. Furthermore, clinicians should consider adenoid hypertrophy as a potential early indicator of scoliosis, warranting timely investigation.

Our study underscores the necessity of a comprehensive approach to scoliosis diagnosis, combining clinical and radiological parameters. Accurate early diagnosis is essential for effective intervention, improved patient outcomes, and the optimization of healthcare resources. Our findings also contribute to the ongoing discussion regarding the utility of chest radiographs in scoliosis assessment. Further research is imperative to refine diagnostic strategies for this prevalent orthopedic condition.

However, this study has notable limitations. First, the absence of lateral vertebral column radiographs prevents the evaluation of sagittal plane spinal deformities, restricting a comprehensive understanding of the spine's three-dimensional distortion. Additionally, the study's retrospective design hinders the acquisition of more extensive clinical data about patients, thus limiting the depth of insight into potential influencing factors. Future prospective studies encompassing a larger patient cohort are essential to gain a more comprehensive perspective. These endeavors promise to shed further light on the interplay between adenoid hypertrophy, hypoxia, and EOS.

## Conclusions

With the heightened incidence of EOS evident among pediatric cohorts undergoing adenoidectomy procedures, this investigation prompts a compelling exploration into the intricate interplay between adenoid hypertrophy, hypoxic states, and the genesis of premature vertebral curvature. This study underscores the imperative nature of prospective investigations aimed at unraveling the multifaceted mechanisms that intricately connect these variables. Such studies offer insightful revelations concerning potential etiological elements and latent pathogenic trajectories that converge to initiate EOS manifestations. Furthermore, due consideration should be given to instigating consultations with pertinent subspecialists when anomalies manifest within lung radiographs of patients scheduled for adenoidectomy, as prospective pathologies may be discernible, necessitating timely interventions to prevent future complications.
